# MiR-425-5p intervenes in autoimmune myocarditis by regulating Treg cell differentiation through NRAS

**DOI:** 10.3389/fcell.2025.1600103

**Published:** 2025-05-13

**Authors:** Shan Zhou, Li Zhang, Xiuyun Duan, Keyu Liu, You Yingnan, Mengjie Ma, Bo Han

**Affiliations:** Department of Pediatric Cardiology, Shandong Provincial Hospital Affiliated to Shandong First Medical University, Jinan, Shandong, China

**Keywords:** miR-425-5p, myocarditis, Treg, NRAS, immunity

## Abstract

**Aim:**

Our Previous research revealed significant differences in exosome-mediated intercellular miR-425a-5p between normal children and those with fulminant myocarditis. We sought to elucidate the molecular underpinnings and functional implications of miR-425a-5p in the context of myocarditis progression.

**Methods:**

Bioinformatics techniques were employed to predict NRAS as the target gene of miR-425a-5p. We constructed a cellular myocarditis paradigm through LPS-mediated provocation of AC16 cardiomyocyte cultures. MiR-425a-5p was overexpressed, and the expressions of NRAS, cell apoptosis, and proinflammatory cytokine profiles, encompassing IL-1β, IL-6, and TNF-α, were comprehensively quantified. An experimental autoimmune myocarditis (EAM) mouse model was created using adeno-associated virus (AAV) for miR-425a-5p overexpression. Comprehensive histopathological analyses were conducted utilizing multiple staining techniques, including hematoxylin-eosin (HE), immunohistochemical, and Masson trichrome methodologies to characterize tissue responses.

**Results:**

The study demonstrated that miR-425a-5p alleviated the inflammatory response in both AC16 cells and EAM mice through NRAS mediation. Single-cell data analysis of cardiac immune cells revealed that miR-425a-5p promoted Treg cell differentiation and improved cardiac function.

**Conclusion:**

MiR-425a-5p plays a crucial role in modulating inflammatory responses in myocarditis, potentially offering a novel therapeutic strategy for managing the disease.

## 1 Introduction

As essential molecular mediators, miRNAs play fundamental roles in modulating gene expression through post-transcriptional mechanisms and RNA interference pathways (Gan et al., 2022). In addition, recent studies have revealed that, apart from the classical downregulation of gene expression, microRNAs have seven non-classical regulatory molecular mechanisms. For example, pri-miRNAs can be translated into peptides, enter the cytoplasm, be recognized as mRNAs by ribosomes, and translated into peptides to perform physiological functions. Also, miRNA can form a RISC with AGO proteins, and in addition to targeting mRNAs for degradation, it can also bind to other functional proteins to exert non-classical regulatory pathways. These non-classical pathways add more complexity and diversity to the functions of microRNAs.

These molecular regulators exert substantial influence on cardiac and vascular pathophysiology, from early development to disease manifestation and potential recovery (Zhou et al., 2018), such as myocardial infarction, hypertension, and atherosclerosis. The study of microRNAs in cardiovascular diseases has opened up new ways to develop diagnostic and treatment strategies (Michell and Vickers, 2016; Kontidou et al., 2023). In cardiovascular diseases, microRNAs have emerged as crucial mediators of controlling many different biological activities, such as maintaining mitochondrial function (Roiz-Valle et al., 2023), inflammation (Emmi et al., 2022), oxidative stress (Ibáñez-Cabellos et al., 2023), apoptosis (Diakos et al., 2010), angiogenesis (Pencheva et al., 2012), and lipid metabolism (Elsakka et al., 2023). By regulating these processes, microRNAs can lead to the development or worsening of cardiovascular diseases. MicroRNAs also have potential to be used as diagnostic (Wang and Zhang, 2020) and therapeutic tools (Poller et al., 2018). Researchers have identified specific microRNAs that are dysregulated in cardiovascular diseases, and these microRNAs could be used as biomarkers for early diagnosis or disease monitoring. Additionally, by using drugs or gene therapies that target microRNAs that are not functioning properly (Kara et al., 2022), we may be able to treat heart and blood vessel problems more effectively in the future (Li and Rana, 2014).

We employed small RNA microarray profiling to explore plasma exosomal miRNA signatures in pediatric fulminant myocarditis (FM). Differentially expressed miRNAs were identified (The complete dataset has been deposited in GEO repository (accession: GSE221090), and miR-425a-5p was selected as the candidate for subsequent investigation. MiR-425a-5p belongs to a group of microRNA molecules, which target messenger RNA transcripts and stop them from making protein (Sameti et al., 2023). Research has found that miR-425 can help prevent some cancers from growing (Zhao et al., 2021), while in others it can promote tumorigenesis (Liu et al., 2020). The role of miR-425 in disease is complex and context-dependent, highlighting the need for further research to fully understand its functions and potential therapeutic applications. It has been identified as a key regulator of diverse fundamental cellular processes, including cell proliferation (Liu et al., 2022), cell migration (Wang et al., 2022), and angiogenesis (Kim et al., 2022). It has also been associated with different health conditions, such as cancer (Jian et al., 2023), heart problems, and disorders affecting the nervous system. This means that when the levels or activity of MiR-425 are altered, it may play a role in driving the onset or advancement of these diseases. Understanding the role of MiR-425 in these conditions is important because it may provide insights into potential therapeutic targets or diagnostic markers for these diseases.

## 2 Materials and methods

### 2.1 Target gene prediction

To predict target genes for miR-425, we input miR-425 into TargetScan and miRDB, take the intersection, and predict target genes. We then used the R package “clusterProfiler, enrichplot, ggplot2, pathview, ggnewscale, DOSE” for KEGG enrichment analysis.

### 2.2 Cell culture treatments and conditions

The human cardiomyocyte (HCM) cell line (AC16), which was procured from Zhong Qiao Xin Zhou Biotechnology (Shanghai, China). Supplementary Document 1 provides the cell line’s certification, including the STR analysis report. The cellular population was cultured in a controlled environment at 37°C. The growth medium consisted of Dulbecco’s Modified Eagle Medium (DMEM; Gibco, CA, United States) enriched with 10% bovine fetal serum (BFS; Gibco) and 5% CO_2_. To induce an inflammatory state, we employed endotoxin derived from gram-negative bacteria (lipopolysaccharide, LPS; Sigma-Aldrich, MO, United States). Following a 24-h post-passage incubation period, the experimental group was subjected to lipopolysaccharide (LPS) stimulation at a final concentration of 10 μg/mL. In parallel, the control cohort received an equivalent volume of sterile physiological saline solution.

### 2.3 Lentiviral transduction

Lentiviral constructs designed for miR-425-5p overexpression and their corresponding non-targeting controls were custom-synthesized by Heshengbio (Qingdao, China). These engineered viral vectors were engineered to constitutively express green fluorescent protein (GFP) as a reporter gene, facilitating visual confirmation of successful transduction. The examined sequences are as follows: 5′-AAUGACACGAUCACUCCCGUUGA-3′ (LV-miR-425-5p), 5′-UUCUCCGAACGUGUCACGU (LV-NC).

### 2.4 Mice and establishment of EAM model

Six-week-old male mice, exhibiting a mean body mass of 18 ± 2 g, were obtained from Vital River Laboratory Animal Technology (Beijing, China). These specific pathogen-free (SPF) animals were maintained in a controlled environment adhering to stringent biosafety protocols. The housing facility was designed to minimize exogenous microbial contamination, ensuring optimal conditions for experimental integrity. All protocols involving animal experiments have been approved by the Ethics Committee of Shandong Provincial Hospital (NSFC: NO.2018-115). Mice received two subcutaneous injections of 250 μg α-MyHC peptide (AnaSpec, AS-62554) on day 0 and day 7. Prior to immunization, the peptide was emulsified with an equal volume of Freund’s complete adjuvant (Sigma-Aldrich, F5881) in a 1:1 ratio (v/v) and homogenized to form a stable milky emulsion. On day 21, mice were intraperitoneal anesthesia with 0.3% Pentobarbital sodium (0.15 mL/10 g) and maintained at physiological temperature. After the completion of echocardiography, the mice were sacrificed by spinal cord dislocation and the specimens were collected (Figure 3A).

### 2.5 RAAV9 treatment

RAAV9-miR-425-5p and rAAV9-NC were synthesized by Genechem Biotechnology (Shanghai, China). Experimental subjects were administered either the miR-425-5p-expressing rAAV9 vector or its negative control counterpart through caudal vein injection. The viral titer for both constructs was standardized at 5.0 × 10^11^ vector genomes per milligram of body weight. A 3-week incubation period followed the viral delivery, subjects were anesthetized via intraperitoneal injection of 0.3% pentobarbital sodium (0.15 mL/10 g), then euthanized by cervical dislocation, after which specimens were collected.

### 2.6 Quantitative PCR (qPCR) analysis

RNA isolation from cellular and tissue specimens was performed using a guanidinium thiocyanate-phenol-chloroform extraction method. The procedure employed TRIzol reagent (Thermo Fisher Scientific, Waltham, MA, United States; catalog number: 15596026) and adhered to the protocol provided by the supplier. Custom oligonucleotide primers targeting specific genes were designed and synthesized by a commercial provider (RiboBio Co., Ltd., Guangzhou, Guangdong, China). Quantitative PCR analysis was conducted on a QuantStudio 5 platform (Applied Biosystems, part of Thermo Fisher Scientific, Waltham, MA, United States) utilizing SYBR Green chemistry for amplicon detection. The reaction mixture contained SYBR Green PCR Master Mix (Applied Biosystems; catalog number: 4309155). Transcript abundance was quantified using the comparative Ct method (also known as the 2^(−ΔΔCt)^ approach). Expression levels were normalized to the endogenous control gene GAPDH.

### 2.7 Western blot

Cellular and tissue-derived protein extracts were obtained through a comprehensive lysis procedure utilizing radioimmunoprecipitation assay (RIPA) buffer, which was strategically fortified with a cocktail of protease inhibitory agents to preserve protein integrity and prevent potential degradation. Protein samples (20 μg) underwent electrophoretic separation via 10% SDS-PAGE and were subsequently electrotransferred onto PVDF membranes. Following a 1-h blocking procedure with 5% non-fat milk in TBST at room temperature, membranes were incubated overnight at 4°C with primary Nras-specific antibodies (1:1,000, Abcam, ab300431, United Kingdom) and primary antibodies again β-actin (1:10,000, Affinity Biosciences, United States). HRP-conjugated secondary antibodies were applied, followed by enhanced chemiluminescence detection and densitometric analysis using ChemiDoc system.

### 2.8 Apoptosis

Apoptosis was assessed via flow cytometry using an Annexin V-PE/7AAD apoptosis kit (BD Biosciences, NJ, United States). Cells were harvested, washed with PBS, and resuspended in Annexin V binding buffer. After staining with Annexin V-PE and 7AAD, samples were incubated in darkness for 15 min at room temperature. Flow cytometric analysis was performed on an ATTUNE NXT (ThermoFisher, Singapore), recording ≥10,000 events. Cells were categorized as viable, early apoptotic, late apoptotic, or necrotic based on Annexin V-PE and 7AAD staining. Cardiac tissue sections (4–5 μm) were deparaffinized, rehydrated, and post-fixed with 4% paraformaldehyde. After permeabilization with 0.1% Triton X-100 and blocking with 5% BSA, sections were incubated with TUNEL reaction mixture (60 min, 37°C). DNase I-treated and TdT-omitted sections served as controls. Nuclei were counterstained with Hoechst 33342, and optional propidium iodide staining distinguished necrotic cells. Images were captured by confocal microscopy, Statistical significance was determined using one-way ANOVA (*p* < 0.05).

### 2.9 Histological staining and immunohistochemistry

Tissue specimens were processed through standard histological techniques. Hematoxylin and eosin (H&E) staining followed conventional protocols. Masson’s trichrome staining involved sequential mordanting, nuclear and cytoplasmic labeling, and collagen visualization using specialized reagents. For immunohistochemistry, sections underwent antigen retrieval, blocking, and primary antibody incubation (1:200, Abcam, ab300431, United Kingdom). Visualization was achieved using biotinylated secondary antibody, avidin-biotin complex, and 3,3′-diaminobenzidine. Sections were counterstained and examined under light microscopy at ×200 and ×400 magnifications. Quantitative analysis was performed using ImageJ software across five random fields, with statistical significance determined by t-test (*p* < 0.05).

### 2.10 Echocardiography echocardiography

On day 21, the mice were subjected to echocardiography using a SiliconWave30 high-frequency ultrasound system (Kolo Medical, Suzhou, China). Mice were intraperitoneal anesthesia with 0.3% Pentobarbital sodium (0.15 mL/10 g) and maintained at physiological temperature. Ejection fraction (EF), Fraction shortening (FS), Left ventricular end diastolic diameter (LVIDd), Left ventricular end systolic diameter (LVIDs) parameters were assessed in M-mode and B-mode from parasternal long-axis views. Data are presented as mean ± SD, Comparisons between groups were made using unpaired Student’s t-test (*p* < 0.05).

### 2.11 The proportion of Treg cells in the blood and spleen

Single-cell suspensions were obtained through selective erythrocyte removal and subsequently immunolabeled with fluorochrome-conjugated antibodies for precise cellular phenotyping. After 30-min incubation on ice following standard protocols. Intracellular Foxp3 staining was performed using specific antibodies. Flow cytometric analysis was conducted on a FACSARIA Fusion (version 10.8, BD Biosciences) to assess the expression. The sequential gating steps: (i) initial lymphocyte selection based on FSC/SSC properties, (ii) CD45^+^ leukocyte gating, (iii) CD3^+^ T cell selection, (iv) CD4^+^ T helper cell gating, and (v) final Foxp3+ Treg identification. The percentage of cells in each category was calculated and Ordinary one-way ANOVA test (*p* < 0.05).

### 2.12 Cytokine quantification

Inflammatory cytokine profiles (TNF-α, IL-1β, IL-6) were measured by enzyme-linked immunosorbent assay (ELISA) using commercially available kits (Elabscience). Statistical significance was determined through two-way ANOVA test (*p* < 0.05).

### 2.13 Analysis of Treg cells in cardiac immune populations via single-cell sequencing

Single-cell RNA sequencing (scRNA-seq) data from the public database GSE142564 were analyzed. This dataset was generated using an experimental autoimmune myocarditis (EAM) model, which corresponds to the original study published by JiangPing Song’s team (Hua et al., 2020). We compared the 0-Day group (GSM4231966) and the 21-day EAM group (GSM4231968) for our analysis. Prior to t-SNE visualization, the data underwent rigorous preprocessing steps, including quality control (e.g., removal of low-quality cells with mitochondrial gene content >5%, genes expressed in <3 cells), normalization, and dimensionality reduction via principal component analysis (PCA). The t-SNE algorithm was applied to the top 10 principal components using the Seurat v4.0, pipeline to visualize cell clusters in a two-dimensional space. Following single-cell analysis, regulatory T cell (Treg) populations were specifically extracted for Gene Ontology (GO) enrichment analysis to identify functional pathways associated with autoimmune myocarditis progression.

### 2.14 Dual-luciferase reporter assay

The NRAS 3′UTR was PCR-amplified from human genomic DNA and inserted into pGL3-basic vector downstream of the luciferase gene. The putative miR-425-5p binding site within NRAS 3′UTR was identified using TargetScan 7.2 and mutated to generate the mutant construct. HEK293FT cells were maintained in DMEM supplemented with 10% FBS and 1% penicillin-streptomycin at 37°C with 5% CO_2_.

HEK293FT cells were seeded in 24-well plates (5 × 10^4^ cells/well) 24 h before transfection. At 70%–80% confluence, cells were co-transfected with 450 ng firefly luciferase reporter plasmid (pGL3-NRAS-3′UTR-WT or pGL3-NRAS-3′UTR-MT), 50 ng pRL-TK Renilla luciferase construct, and miR-425-5p mimics or negative control (NC) at 50 nM final concentration using Lipofectamine 3000 (Invitrogen). Four experimental groups were established (Gan et al., 2022): WT+NC (Zhou et al., 2018), WT+miR-425-5p mimics (Michell and Vickers, 2016), MT+NC, and (Kontidou et al., 2023) MT+miR-425-5p mimics. Transfections were performed according to manufacturer’s protocol by combining DNA-lipid complexes (formed in Opti-MEM) with miRNA solutions. Five biological replicates were prepared per group.

At 48 h post-transfection, cells were lysed with Passive Lysis Buffer. Firefly and Renilla luciferase activities were measured sequentially using the Dual-Luciferase Reporter Assay System on a GloMax detection system. Relative luciferase activity was calculated as firefly/Renilla ratio and normalized to the WT+NC group (set as 100%). Statistical analyses were performed using one-way ANOVA followed by Tukey’s test in GraphPad Prism 9.0. *P* < 0.05 was considered statistically significant. Data are presented as mean ± SD from five independent replicates.

## 3 Results

### 3.1 Targeted regulation of NRAS by miR-425-5p

**FIGURE 1:**
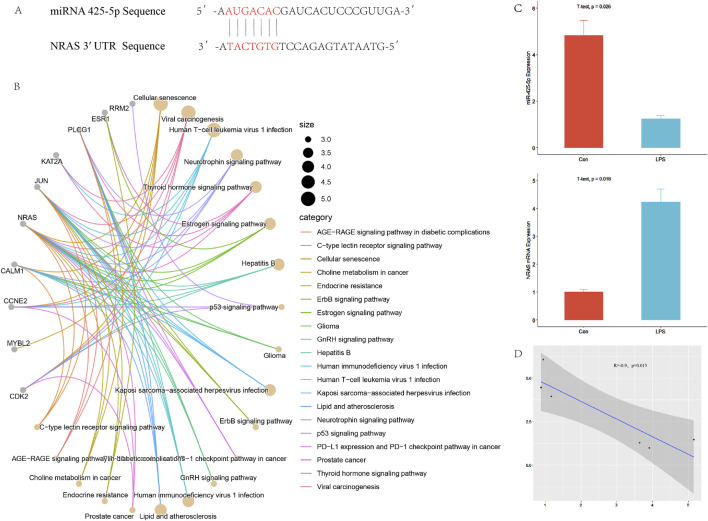
Targeted regulation of NRAS by miR-425-5p. **(A)** The complementary binding site between the 3′UTR of NRAS and the seed sequence of miR-425-5p. **(B)** Prediction of target genes and pathway enrichment analysis of miR-425-5p. **(C, D)** The expression of miR-425-5p and NRAS in the inflammatory model of LPS-stimulated AC16 cell line. There is a correlation between miR-425-5p and NRAS (Pearson correlation analysis, n = 3, R = −0.9, *p* = 0.015).

We predicted 247 target genes using TargetScan and miRDB. Subsequently, we performed KEGG enrichment analysis using R packages, resulting in Figure 1B. In Figure 1A, miR-425-5p binding regions within the NRAS 3′ UTR. Expression levels of miRNA-425-5p and Nras mRNA were assessed in both control and LPS-treated groups. A negative correlation was observed between these two factors, as illustrated in Figures 1C, D.

### 3.2 The role of miR-425-5p in model of AC16 cells

**FIGURE 2:**
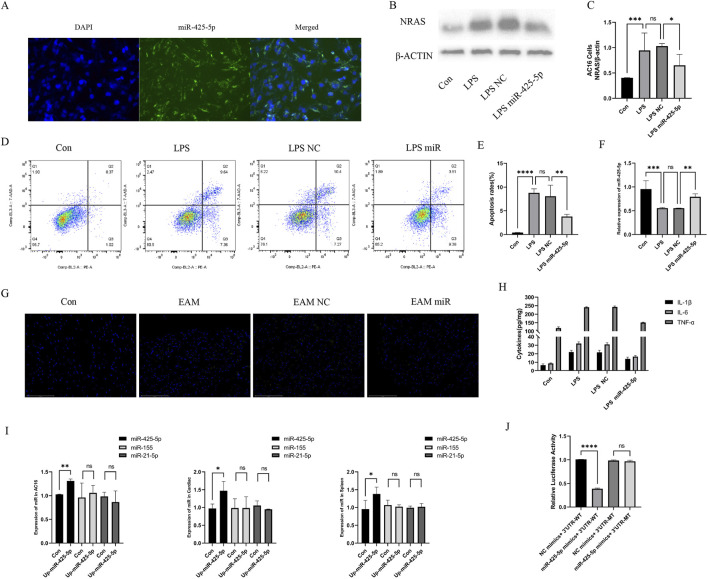
The role of miR-425-5p in model of AC16 cells and EAM model. **(A)** MiR-425-5p was successfully transfected into AC16 cells. **(B, C)** Ex*p*ression and quantitative analysis of NRAS in AC16 cells. **(D, E)** The impact of miR-425-5p on apoptosis in AC16 cells and quantitative analysis. **(F)** Expression of miR-425-5p in AC16 cells. **(G)** Apoptosis of cardiac specimens in the EAM model (Scale bar: 125 μm). **(H)** ELISA of IL-1β, IL-6, and TNF-α in the supernatant of the AC16 cell model. **(I)** After overexpression of mirna-425-5p, the expression of mir-425-5p, miR-155, and mir-21-5p was detected in AC16 cells, mouse cardiac, and spleen. **(J)** MiR-425-5p directly targets NRAS 3′UTR. Luciferase activity was significantly reduced in cells co-transfected with miR-425-5p mimics and wild-type NRAS-3′UTR reporter, but remained unchanged with the mutated binding site reporter. Data are presented as mean ± SD (n = 3 biologically independent samples). *p < 0.05; **p < 0.01; ***p < 0.001 (Student’s t-test).

To confirm the successful transfection of miRNA-425-5p into AC16 cells, we examined the fluorescence carried by miRNA-425-5p Lentivirus vectors in AC16 cells, as illustrated in Figure 2A. We also detected the difference between the overexpression group and the con group and evaluated the overexpression efficiency in Figure 2I. In the LPS-stimulated AC16 cell model, we assessed the expression levels of Nras protein and β-ACTIN across four experimental groups: control, LPS-treated, LPS with negative control, and LPS with miR-425-5p overexpression. Quantitative analysis of these protein levels is presented in Figures 2B, C. MiR-425-5p’s influence on AC16 apoptosis was assessed and quantified in Figures 2D, E. Transcript levels were characterized in AC16 cellular model, as depicted in Figure 2F. Cardiac tissue apoptosis in the EAM model was visualized (Scale bar: 125 μm) in Figure 2G. Inflammatory cytokines IL-1β, IL-6, and TNF-α were quantified in AC16 cell supernatants via ELISA in Figure 2H.

### 3.3 Amelioration of myocarditis by miR-425-5p in EAM model

**FIGURE 3:**
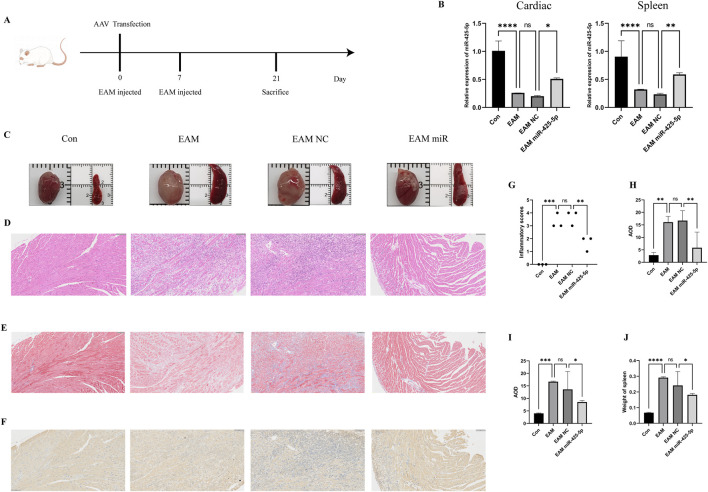
Construction of the EAM model and the improvement of myocardial inflammation by miR-425-5p. **(A)** Construction of the EAM model mediated by AAV carrying miR-425-5p. **(B)** Expression of miR-425-5p in the heart and spleen of EAM. **(C)** Photographs of mice hearts and spleens. **(D, G)** HE staining and inflammation scoring of mice hearts (Scale bar: 100 μm). **(E, H)** Masson’s staining and AOD (Average Optical Density) scoring of mice hearts (Scale bar: 100 μm). **(F, I)** Immunohistochemistry of NRAS in mice hearts and AOD (Average Optical Density) scoring (Scale bar: 100 μm). **(J)** The weight of the mice spleen. Data are presented as mean ± SD (n = 3 biologically independent samples). *p < 0.05; **p < 0.01; ***p < 0.001 (Student’s t-test).

We detected the expression of mirna-425-5p, mirna-155, mirna-21a-5p in heart and spleen of overexpression group and con group, and evaluated the overexpression efficiency and specificity in Figure 2I. Transcript levels in EAM-affected heart and spleen tissues in Figure 3B. Representative images of heart and spleen from mice in Figure 3C. HE staining and inflammation scoring of heart tissues in Figures 3D, G. Masson’s trichrome staining and AOD quantification in heart tissues in Figures 3E, H (scale bar: 100 μm). Immunohistochemical analysis of NRAS expression in heart tissue with AOD scoring in Figures 3F, I (scale bar: 100 μm). (J) Spleen weight measurements in mice in Figure 3J.

### 3.4 Changes in the expression of NRAS, echocardiography, proportion of Treg cells, and serum ELISA in EAM model

**FIGURE 4:**
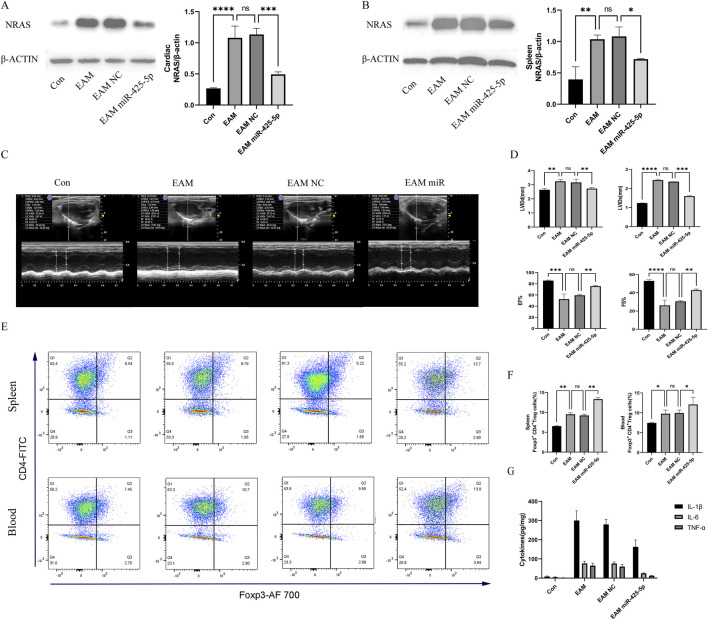
Changes in the expression of NRAS, echocardiography, proportion of Treg cells, and serum ELISA in mice. **(A, B)** Expression and quantitative analysis of NRAS in the heart and spleen of mice. **(C, D)** Changes in echocardiography and quantitative analysis of LVIDd, LVIDs, EF%, and FS% in mice. **(E, F)** Quantitative analysis of the proportion of Treg cells in the spleen and blood of mice (represented by CD4+FITC and Foxp3+AF700), as well as their quantification. **(G)** ELISA for IL-1β, IL-6, and TNF-α in mouse serum. Data are presented as mean ± SD (n = 3 biologically independent samples). *p < 0.05; **p < 0.01; ***p < 0.001 (t-test).

NRAS molecular expression and quantitative assessment in murine cardiac and splenic tissues, illustrated in Figures 4A, B. Changes in echocardiography and quantitative analysis of LVIDd, LVIDs, EF%, and FS% in mice in Figures 4C, D. Regulatory T cell (Treg) frequency assessment in murine splenic and peripheral blood compartments (represented by CD4+FITC and Foxp3+AF700), as well as their quantification in Figures 4E, F. ELISA for IL-1β, IL-6, and TNF-α in mouse serum in Figure 4G.

### 3.5 Proportion analysis of Treg cells in cardiac immune populations

**FIGURE 5:**
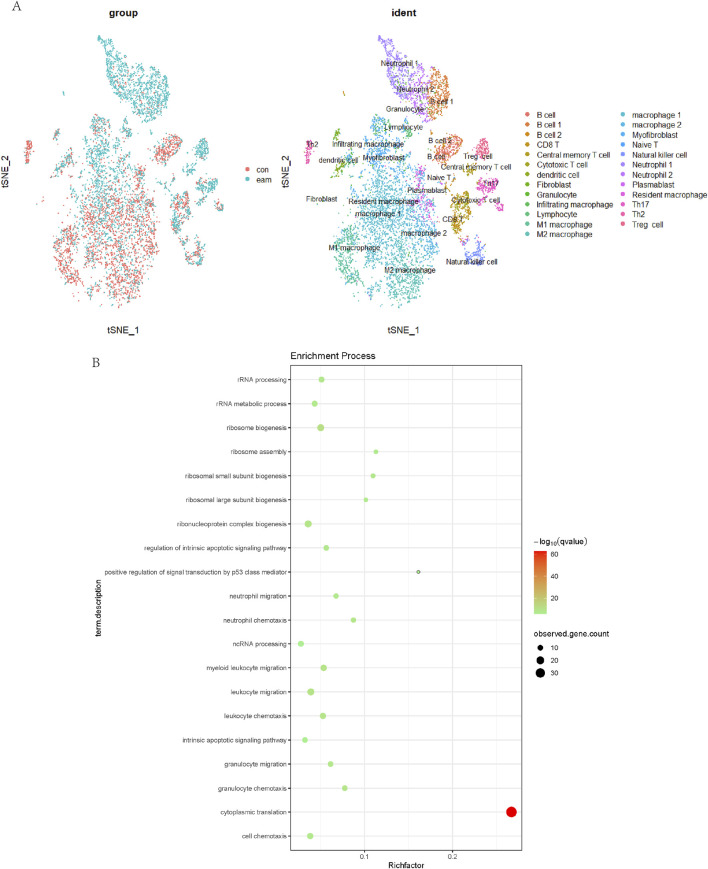
Analysis of Treg Cells in Cardiac Immune Populations. The t-SNE plot **(A)** highlighted the most pronounced differences between EAM and CON groups. Gene Ontology (GO) pathway enrichment analysis **(B)** indicated highly active cytoplasmic translation pathways in Treg cells.

Single-cell data analysis revealed significant differences between EAM and CON groups in macrophage, neutrophil, and Treg cell clusters. The t-SNE plot highlighted the most pronounced differences in Treg cells, as shown in Figure 5. Gene Ontology (GO) pathway enrichment analysis indicated highly active cytoplasmic translation pathways in Treg cells.

### 3.6 Dual-luciferase reporter assay

Transfection of miR-425-5p mimics significantly reduced luciferase activity of the NRAS-3′UTR-WT reporter compared to the negative control in Figure 2J. In contrast, miR-425-5p mimics had no significant effect on the NRAS-3′UTR-MT reporter, confirming that mutation of the binding site abolished miR-425-5p-mediated repression. Consistent Renilla luciferase activities across all experimental groups confirmed uniform transfection efficiency. These data provide strong evidence that miR-425-5p directly targets the NRAS 3′UTR through the identified binding site, thus functioning as a negative regulator of this important gene.

## 4 Discussion

We demonstrated that miR-425-5p alleviates myocarditis through the regulation of NRAS and subsequent modulation of TREG cell differentiation. Our findings reveal a novel mechanism by which miR-425-5p functions as a potential therapeutic target in myocarditis treatment. First, MiR-425-5p levels were downregulated in myocarditis comparative analysis of healthy controls, suggesting its potential involvement in disease progression. This observation aligns with previous studies that have reported altered microRNA expression profiles in various cardiovascular diseases (Zhang X. et al., 2023; Li et al., 2021). The inverse correlation between miR-425-5p levels and disease severity further supports its protective role in myocarditis. Mechanistic studies demonstrated that NRAS expression was inversely correlated with miR-425-5p levels. NRAS, belonging to the RAS small GTPase signaling network, has been previously associated with immune response regulation (Rougerie and Delon, 2012). The identification of this interaction provides new insights into the molecular pathways governing myocarditis progression.

Notably, we found that miR-425-5p-mediated suppression of NRAS significantly enhanced TREG cell differentiation. Treg cells play a vital role in maintaining immune homeostasis (Fisher and Sennikov, 2025). Our research subjects are BALB/c mice, which are frequently utilized in immunological studies and exhibit a slightly higher proportion of Treg cells compared to other mouse strains (Godoy et al., 2020). The observed increase in TREG cell population following miR-425-5p overexpression suggests a potential immunomodulatory mechanism through which this microRNA exerts its protective effects. Furthermore, *In vivo* experiments revealed that miR-425-5p markedly attenuated inflammatory cell infiltration and enhanced cardiac performance in myocarditis. These results corroborate existing research underscoring the significance of Treg cells in regulating immune responses within cardiovascular pathologies (Wang et al., 2024).

The therapeutic potential of miR-425-5p is particularly promising given its specific targeting of the NRAS/TREG axis. Unlike broad-spectrum immunosuppressive treatments, this approach may offer a more targeted strategy with potentially fewer side effects. However, additional research is required to refine delivery strategies and assess comprehensive long-term safety parameters. It is worth noting that while strong evidence for the protective role of miR-425-5p in myocarditis, several limitations should be addressed in future research. First, the exact mechanism by which NRAS regulation affects TREG cell differentiation requires further investigation. Additionally, the potential effects of miR-425-5p on other immune cell populations and inflammatory mediators should be explored.

Recent research demonstrates that microRNAs (miRNAs) play crucial regulatory roles in myocarditis pathogenesis by modulating diverse immune pathways. Multiple miRNAs have emerged as key mediators of inflammatory processes in cardiac tissue during myocarditis development and progression.

MiR-155 appears to be a critical regulator of T cell responses in experimental autoimmune myocarditis (EAM). Significantly upregulated in both cardiac tissue and CD4^+^ T cells during EAM, miR-155 promotes disease progression by driving an imbalance between pro-inflammatory Th17 cells and immunosuppressive Treg cells. This imbalance results from enhanced Th17 cell development and their increased resistance to Treg-mediated suppression. Importantly, miR-155 inhibition attenuates disease severity, reduces cardiac injury, and decreases dendritic cell secretion of Th17-polarizing cytokines, suggesting therapeutic potential (Yan et al., 2016). Another important regulator, miR-21a-5p, demonstrates significant upregulation in EAM models. Silencing miR-21a-5p reduces pro-inflammatory cytokine expression (TNFα, IL-6) and collagen production *in vitro*, while *in vivo* treatment with antagomiR-21a-5p formulated in polymeric nanoparticles attenuates myocardial inflammation, fibrosis, and adverse cardiac remodeling. These findings highlight miR-21a-5p as another potential therapeutic target (Mirna et al., 2022). Additionally, investigations into myocardial injury associated with dermatomyositis have identified miR-146a-5p as significantly elevated in cases with cardiac involvement. This miRNA appears to regulate genes enriched in type I interferon signaling pathways (IFIT3, OAS3, ISG15, and RSAD2), which correlate with increased M2 macrophage infiltration in both dermatomyositis and myocarditis (Zhang Y. et al., 2023).

Collectively, these findings demonstrate that miRNAs influence myocarditis through multiple immune mechanisms: modulating T cell subset balance, regulating cytokine production, affecting macrophage function, and influencing interferon signaling pathways. The therapeutic potential of miRNA-based interventions is supported by experimental evidence showing disease attenuation through specific miRNA targeting. Further research is needed to translate these findings into clinical applications.

## 5 Conclusion

This study demonstrates that miR-425a-5p plays a crucial protective role in myocarditis through multiple mechanisms. Our findings reveal that miR-425a-5p directly targets NRAS and significantly attenuates inflammatory responses both *in vitro* and *in vivo*. Specifically, we observed that miR-425a-5p overexpression effectively reduced pro-inflammatory cytokine production (IL-1β, IL-6, and TNF-α) in LPS-stimulated AC16 cardiomyocytes and ameliorated cardiac inflammation in the EAM mouse model. Moreover, miR-425a-5p promoted Treg cell differentiation and cardiac performance was enhanced in EAM, confirmed through ultrasound imaging. The identification of the miR-425a-5p/NRAS axis provides novel molecular perspectives on myocarditis pathogenesis and potential therapeutic interventions. These findings highlight miR-425a-5p Representing a novel therapeutic strategy in fulminant myocardial inflammation. Future studies should focus on developing miR-425a-5p-based therapeutic approaches and investigating their clinical applications in myocarditis treatment.

## Data Availability

Publicly available datasets were analyzed in this study. This data can be found here: https://www.ncbi.nlm.nih.gov/geo/query/acc.cgi?acc=GSE142564.
